# Integrated Genome-Wide Analysis of an Isogenic Pair of *Pseudomonas aeruginosa* Clinical Isolates with Differential Antimicrobial Resistance to Ceftolozane/Tazobactam, Ceftazidime/Avibactam, and Piperacillin/Tazobactam

**DOI:** 10.3390/ijms21031026

**Published:** 2020-02-04

**Authors:** Weihua Huang, Joelle El Hamouche, Guiqing Wang, Melissa Smith, Changhong Yin, Abhay Dhand, Nevenka Dimitrova, John T. Fallon

**Affiliations:** 1 Department of Pathology, New York Medical College, Valhalla, NY 10595, USA; hank.wang@wmchealth.org (G.W.); changhong_yin@nymc.edu (C.Y.); fallonj19@ecu.edu (J.T.F.); 2 Department of Biomedical Engineering, Stony Brook University, Stony Brook, NY 11790, USA; joellelhamouche@gmail.com; 3 Department of Pathology and Clinical Laboratories, Westchester Medical Center, Valhalla, NY 10595, USA; 4 Department of Genetics and Genomic Sciences, Icahn School of Medicine at Mount Sinai, New York, NY 10029, USA; melissa.smith@mssm.edu; 5 Department of Medicine, New York Medical College, Valhalla, NY 10595, USA; Abhay.Dhand@wmchealth.org; 6 Philips Research North America, Cambridge, MA 02141, USA; nevenka.dimitrova@philips.com

**Keywords:** *Pseudomonas aeruginosa*, whole-genome sequencing (WGS), epigenetic profiling, RNA sequencing (RNAseq), antimicrobial resistance, ceftolozane/tazobactam (C/T), ceftazidime/avibactam (CZA), piperacillin/tazobactam (P/T)

## Abstract

Multidrug-resistant (MDR) *Pseudomonas aeruginosa* is one of the main causes of morbidity and mortality in hospitalized patients and the leading cause of nosocomial infections. We investigated, here, two MDR *P. aeruginosa* clinical isolates from a hospitalized patient with differential antimicrobial resistance to ceftazidime/avibactam (CZA), ceftolozane/tazobactam (C/T), and piperacillin/tazobactam (P/T). Their assembled complete genomes revealed they belonged to ST235, a widespread MDR clone; and were isogenic with only a single nucleotide variant, causing G183D mutation in AmpC β-lactamase, responsible for a phenotypic change from susceptible to resistant to CZA and C/T. Further epigenomic profiling uncovered two conserved DNA methylation motifs targeted by two distinct putative methyltransferase-containing restriction-modification systems, respectively; more intriguingly, there was a significant difference between the paired isolates in the pattern of genomic DNA methylation and modifications. Moreover, genome-wide gene expression profiling demonstrated the inheritable genomic methylation and modification induced 14 genes being differentially regulated, of which only *toxR* (downregulated), a regulatory transcription factor, had its promoter region differentially methylate and modified. Since highly expressed *opdQ* encodes an OprD porin family protein, therefore, we proposed an epigenetic regulation of *opdQ* expression pertinent to the phenotypic change of *P. aeruginosa* from resistant to susceptible to P/T. The disclosed epigenetic mechanism controlling phenotypic antimicrobial resistance deserves further experimental investigation.

## 1. Introduction

*Pseudomonas aeruginosa* is one of the leading causes of nosocomial infections with an estimated 32,600 healthcare-associated infections and 2700 deaths per year in the United States [1]. Since the organism is capable of acquiring resistance to almost all antibiotics commonly used against it [2], treatment options for multidrug-resistant (MDR) *P. aeruginosa* are very limited. MDR *P. aeruginosa* is, thus, categorized as a serious threat to public health by the Centers for Disease Control and Prevention (CDC) [1]. Characterizing *P. aeruginosa* clinical lineages and determining how they adapt and acquire multiple antimicrobial resistance elements are, hence, of high scientific and clinical importance.

The first genome sequence of *P. aeruginosa* PAO1 strain was published in 2000 [3]. As of December 2019, there are 5027 genome assembly and annotation reports of *P. aeruginosa* isolates publicly available at the National Center for Biotechnology Information (NCBI) database, 212 of which are complete genomes. *P. aeruginosa* is among the largest of bacterial genomes, with a median size of 6.6 mega base pairs (bp) and a median GC content of 66.2%. On the one hand, the repertoire of *P. aeruginosa* genes, foundational to respond and adapt to diverse environments, are substantially conserved with a high proportion of regulatory genes and networks observed in other known bacterial genomes [3,4,5,6]. On the other hand, analyses of *P. aeruginosa* complete genomes revealed remarkable plasticity [4,5]. In fact, *P. aeruginosa* genome often carries many diverse mobile genetic elements responsible for horizontal gene transfer, which include plasmids, prophages, integrative and conjugative elements, insertion sequences, and transposons. 

Restriction-modification (RM) system is one of prokaryotic key immune systems protecting cells from invading DNA by methylating endogenous DNA and cleaving non-methylated foreign DNA [7]. As parts of a RM system, restriction enzyme (REase) cleaves DNA and methyltransferase (MTase) acts in concert to methylate DNA. There are three forms of DNA methylation, N6-methyladenine (m6A), N4-methylcytosine (m4C), and N5-methylcytosine (m5C). The recent introduction of new sequencing technologies allows direct mapping of all these major DNA modifications. In particular, single-molecule real-time (SMRT) sequencing enables detection of m5C modestly in sensitivity, but both m6A and m4C with a high degree of accuracy and sensitivity [8]. DNA methylation in bacteria, mediated by MTases, has been found to play various important roles in regulation of gene expression, virulence, pathogen-host interaction, as well as antimicrobial resistance [7,9], and thus has gained increasing attention. As shown in eukaryotes, such epigenetic DNA modification can support long-lasting downstream responses in prokaryotes. To date, more than 2000 bacterial and archaeal methylomes have been profiled and are currently available in the centralized REBASE [10]. Moreover, the recent profiling of PAO1 methylome has revealed a conserved sequence motif targeted by a Type I RM system, which is encoded by accessory genes not in the core genome of *P. aeruginosa* [11]. 

Owing to its large genome, *P. aeruginosa* has an extraordinary capacity to develop resistance against a wide range of antimicrobials through various molecular mechanisms. These molecular mechanisms, stemming from the existence of intrinsic resistance genes (such as multidrug efflux systems), mutation or loss of intrinsic genes (such as *amp*C and *opr*D), or acquisition of extrinsic resistance genes from horizontal gene transfer, are often simultaneously present in clinical isolates, making them notoriously MDR [2,6,12]. Additionally, the increasing use of antimicrobials have resulted in dramatically increasing prevalence of nosocomial and chronic infections of MDR *P. aeruginosa*. In this study, we performed whole-genome sequencing and RNA sequencing (RNAseq) on a pair of *P. aeruginosa* clinical isolates collected from a patient and attempted to reveal the following: (1) confirmation of whether the paired isolates were isogenic, (2) identification of the genetic and epigenetic elements responsible for their MDR, and (3) identification of the genetic and epigenetic alterations causing their different phenotypes in antimicrobial susceptibility.

## 2. Results

A hospitalized 73-year-old female with diabetes, morbid obesity, chronic respiratory failure s/p tracheostomy, and extensive antibiotic exposure in past was treated for MDR *P. aeruginosa* infection, with ceftolozane/tazobactam (C/T) for 11 days; 20 days later, with ceftazidime/avibactam (CZA) for three weeks. In routine clinical tests during the CZA treatment, a pair of *P. aeruginosa* isolates, designated PB367 and PB350, were recovered from tracheal aspirate and sputum specimens, respectively, with an interval of two weeks. Antimicrobial susceptibility testing demonstrated that both isolates were MDR, nonsusceptible to 11 of 14 antimicrobial drugs analyzed (Table 1). Notably, the pair of isolates exhibited differential susceptibility to CZA, C/T, and Piperacillin/Tazobactam (P/T). PB367 was resistant to P/T with a higher minimum inhibitory concentration (MIC), but susceptible to both CZA and C/T. In contrast, PB350 became resistant to both CZA and C/T with higher MICs, but susceptible to P/T.

**Figure 1:**
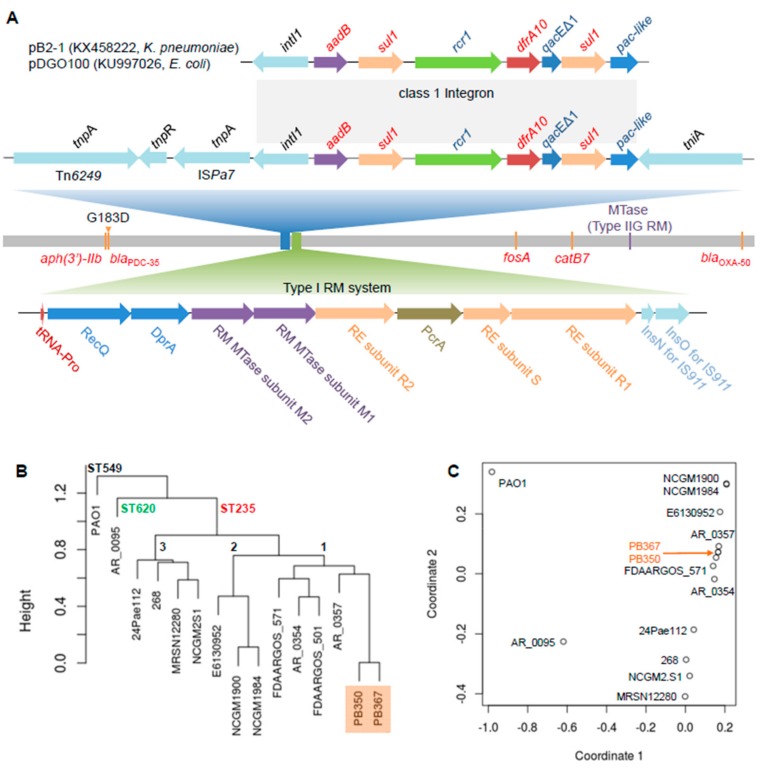
Comparative genome analysis of *P. aeruginosa* isolates. (**A**) Schematic genome of the paired *P. aeruginosa* isolates, PB367 and PB350. Genomic mutation C931,543T (G183D) in β-lactamase gene (*bla*_PDC-35_) from PB367 to PB350 is indicated in the chromosome, along with other antimicrobial resistance genes (*aph*(3’)-IIb, *fos*A, *cat*B7, and *bla*_OXA-50_) and a gene encoding a putative Type IIG restriction-modification (RM) methyltransferase (MTase) targeting a specific motif AAG^m6^AYC. Top: A unique class 1 integron in *P. aeruginosa* with multidrug resistance (MDR) genes, 100% identical to the ones found in plasmids of *K. pneumoniae* and *E. coli*, is located among multiple mobile genetic elements, Tn*6249*, resolvase, IS*Pa7*, and *tni*A. Bottom: A unique Type I RM system comprised of restriction enzymes (RE, subunits R and S) and modification MTases (M1 and M2) putatively targeting a bipartite and asymmetric motif CT^m6^AC(N)_5_GGG/C^m4^CC(N)_5_GTAG; (**B**) Phylogeny tree of 15 *P. aeruginosa* isolates, mainly ST235, from kWIP hierarchical clustering using the complete linkage method. PAO1 belonged to ST549, AR_0095 belonged to ST620, and three main sub-ST235 clusters (1–3) are demonstrated; (**C**) Relatedness overview of 15 *P. aeruginosa* isolates in a plot of metric multidimensional scaling (MDS).

By using Pacific Biosciences (PacBio) long-read and Illumina short-read sequencing, we de novo assembled complete genomes of PB367 and PB350, followed by manually polishing. We found the paired isolates were isogenic, sharing the same genome sequence, except for a single nucleotide variant C931,543T. The mutation was non-synonymous in *bla*_PDC-35_ gene and led to G183D alteration in a class C β-lactamase (AmpC), which was responsible for PD350 resistance to CZA and C/T [13,14,15]. The shared genome had a chromosome size of 6,752,906 bp with a GC content of 66.2%, comprising about 6243 coding genes, 63 transfer RNAs (tRNAs), and 12 rRNAs. The chromosome contained multiple antimicrobial resistance genes (Figure 1A), including species-intrinsic *aph*(3')-IIb, *bla*_PDC-35_ (*amp*C), *cat*B7, *fos*A, and extrinsic *bla*_OXA-50_, which confer resistance to aminoglycosides, β-lactams, phenicols, and fosfomycin. In addition, both PB367 and PB350 carried a unique MDR integron in the chromosome, which contained resistance genes, *aad*B and *dfr*A10, and two copies of *sul*1 (Figure 1A), conferring resistance to aminoglycosides, trimethoprim, and sulphonamides. Carrying integrase *int*I1, the mobile genetic element is defined as a class 1 integron and has previously been found solely in plasmids, *Klebsiella pneumoniae* pB2-1 (KX458222) [16] and *Escherichia coli* pDGO100 (KU997026) [17]. Their completely identical sequences imply the occurrence of horizontal gene transfer between plasmids, between plasmid and chromosome, and between species. Notably, the integron in *P. aeruginosa* chromosome was flanked by a transposase *tniA* at one end and three genetic mobile elements, Tn*6249*, *tnp*R, and IS*Pa7*, at the other end (Figure 1A).

The paired isolates belong to ST235, the most prevalent of so-called international, high-risk, and widespread *P. aeruginosa* clones associated with poor clinical outcomes, in part due to multi- and high-level antimicrobial resistance [18]. Of 189 *P. aeruginosa* complete genomes publicly available (as of May 2019), 13 of which, including PB367 and PB350, belonged to ST235. Their phylogeny tree and strain relatedness are shown in Figure 1B,C, in which strains PAO1 (ST549) and AR_0095 (ST620) are used as controls in the comparison and three main sub-ST235 clusters (1–3) are observed. Notably, three NCGM isolates (2.S1, 1900, and 1984) in two subclusters were from Japan, strain 24Pae112 from Columbia, strain E6130952 from Canada, and the others were from the United States.

**Figure 2:**
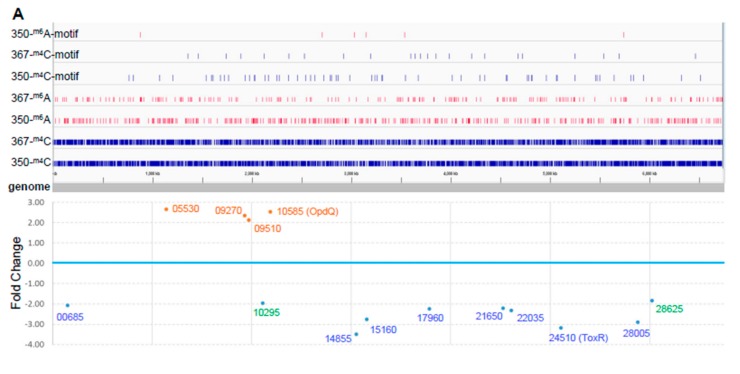

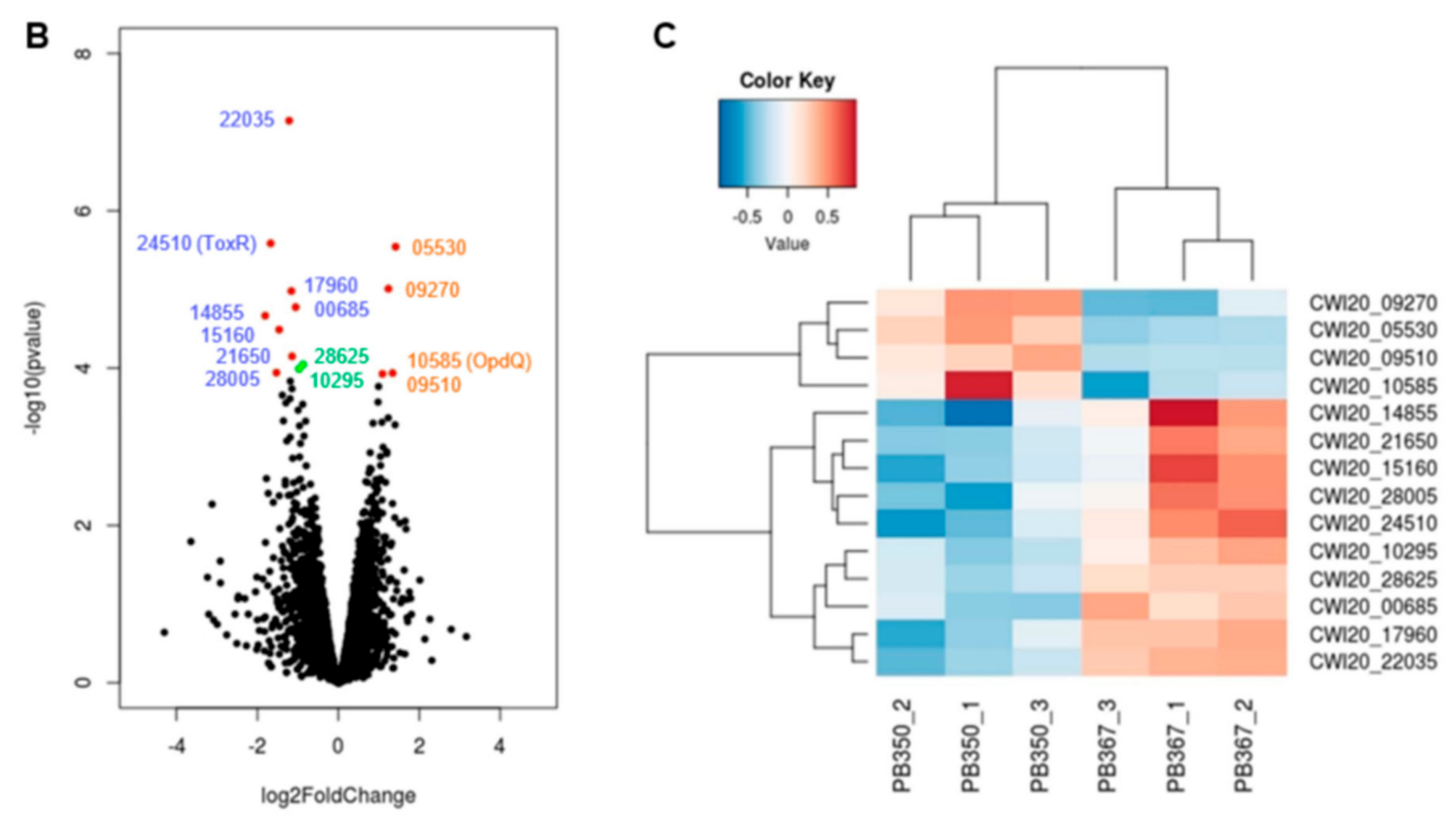
Comparative epigenome and transcriptome analysis of *P. aeruginosa* isolates. (**A**) Genome-wide differential methylation and gene expression in the paired *P. aeruginosa* isolates, PB367 and PB350. Top: Differentially methylated adenosine (m6A, in red) and cytosine (m4C, in blue) in PB367 and PB350, within or without sequence motif (refer to Table 2), and their distribution throughout the whole genome. Bottom: 14 genes identified with significantly differential expression (adjusted *p*-value < 0.05) and their particular loci at the genome. Upregulated genes in PB350 are labeled in brown, downregulated in blue, fold change less than 2 are in green; (**B**) Volcano plot of RNAseq for identification of 14 genes with significantly differential expression; (**C**) Heatmap of genes with significantly differential expression in triplicated samples. The color key represents the amount by which each gene deviates in a particular sample from the gene’s average across all the samples.

In addition to obtaining long reads, PacBio SMRT sequencing allows direct detection of modified nucleotides in the DNA template [19]. Thus, we explored genome-wide DNA methylation and other modifications in the paired isolates (Table 2). We found more DNA methylations and other DNA modifications in PB350 as compared with PB367. While the majority of m6A (8659, > 95%) was shared between the two isolates, most of m4C and other modifications were distinct in either PB367 (71.4% for m4C and 89.4% for other modifications) or PB350 (74.0% for m4C and 90.1% for the others). The genome-wide distribution of these differentially methylated nucleotides, m6A and m4C, within and without sequence motif, is demonstrated in Figure 2A. The result indicates that the paired isolates have their own distinct epigenomic landscape, likely due to their different sources and different growth environments. 

In both isolates, we detected the following two methylation motif sequences (Table 2): AAG^m6^AYC for Type IIG RM system and bipartite, asymmetric CT^m6^AC(N)_5_GGG/C^m4^CC(N)_5_GTAG for Type I RM system. By searching REBASE, we found motif AAG^m6^AYC, along with GGC^m6^AGC, was previously unique in *Meiothermus silvamus* DSM 9946 [8] and, correspondingly, there were two putative Type IIG REases or m6A DNA MTases in two plasmids, one in pMESIL01 (locus tag Mesil_3351 and protein ID ADH65163) and another in pMESIL02 (locus tag Mesil_3646). Our BLAST protein sequence search revealed that Mesil_3351, not Mesil_3646, had a homologous protein in *P. aeruginosa* (PB367 locus tag CWI20_26820, protein ID WP_023098846, protein identity 39%, RM MTase in Figure 1A), suggesting that Mesil_3351 and CWI20_26820 are a putative Type IIG MTase specific for motif AAG^m6^AYC, whereas Mesil_3646 is putatively specific for GGC^m6^AGC. Bipartite motif CT^m6^AC(N)_5_GGG/C^m4^CC(N)_5_GTAG was previously unique in *P. aeruginosa* AR_0095, with two putative DNA MTases identified in a complex, multi-subunit Type I RM system, along with two REases (Figure 1A). One MTase appeared responsible for m6A methylation with a high rate, ~100% in both isolates; and the other for m4C methylation with a relatively lower rate, 90.2% in PB367 and 95.3% in PB350 (Table 2). Notably, the putative Type II RM MTase (CWI20_26820) was conserved only in sub-ST235 clusters 1 and 2, not in subcluster 3; and the whole Type I RM system (~16 kb in length) was conserved in all ST235 strains tested and was recognized as Block #2 of 22 ST235-specific genes [18].

**Figure 3:**
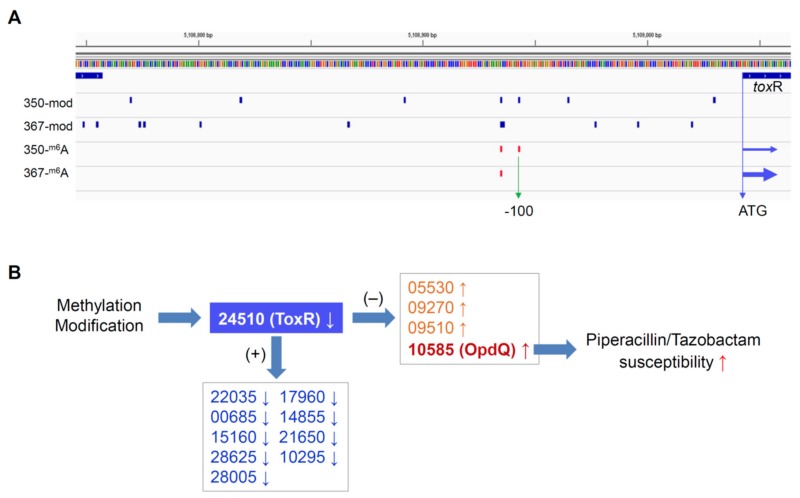
Hypothesized epigenetic regulation mechanism of *P. aeruginosa* antimicrobial resistance. (**A**) Methylation and modification in the promoter of ToxR in isolates PB367 and PB350, an extra m6A site was found in PB350 at −100 (vertical green arrow) flanking from the translation start site (horizontal blue arrows) of ToxR, in addition to differential modifications as compared with PB367; (**B**) Methylation and/or modification cause ToxR lower expression in PB350, which leads to higher expression of OpdQ and, furthermore, PB350 susceptible to Piperacillin/Tazobactam. +, positive transcription regulation; −, negative transcription regulation.

To investigate whether differential DNA methylation and modification affected gene expression in the paired isolates, we further performed genome-wide gene expression analysis using RNAseq. We identified 14 differentially expressed genes in PB350 as compared with PB367, of which four genes were upregulated and ten genes downregulated (Supplementary Table S1 and Figure 2). No significant change was found in the expression of cephalosporinase AmpC, carbapenem porin OprD, or any membrane proteins of multidrug efflux systems that are well-known factors of *P. aeruginosa* antimicrobial resistance mechanisms (Supplementary Table S1) [2,20,21]. Of the 14 genes with altered expression we found the following: Only one encoded regulatory protein with decreased expression (CWI20-24510, homolog of ToxR, PA0707); two were membrane-associated proteins, i.e., CWI20-15160 with reduced expression (homolog of PvdR and PA2389) and CWI20-10585 with increased expression (homolog of OpdQ, PA3038); one was tRNA-Asp; and the remaining were metabolic proteins. Despite the vast epigenetic discrepancy between PB367 and PB350 (Table 2 and Figure 2A), we found few differences of methylation or modification in the promoter regions of these 14 genes, except *tox*R (CWI20-24510) that had an extra m6A methylation and additional differential modifications in PB350 (Figure 3A). This extra m6A methylation was located at -100, upstream of *tox*R translation start site, not in the two motifs of either Type I or Type IIG RM system. We, thus, suspect that ToxR is a positive regulator of the other nine genes downregulated in PB350 and a negative regulator of four genes upregulated in PB350. Since CWI20-10585 (OpdQ) is a member protein of the OprD porin family and since loss or decrease of OprD develops resistance to imipenem and meropenem [2,21,22,23], we further hypothesize that the higher expression of OpdQ in PB350 is most likely responsible for its susceptibility to P/T (MIC = 16 mg/L), changed from resistant PB367 (MIC > 64 mg/L). In conclusion, we propose a potential epigenetic regulatory mechanism for *P. aeruginosa* acquiring resistance to P/T (Figure 3B). 

## 3. Discussion

Integrated profiling of genome, epigenome, and transcriptome provides a global and comprehensive view for functional study. Here, we adopted it to analyze an isogenic pair of *P. aeruginosa* clinical isolates with differential antimicrobial resistance, and found certain advantages. First, by cross-examination of sequence alignment and mapping, the de novo assembled complete genomes are polished more accurately, with less errors; and the paired comparison allows identification of genomic and epigenomic alterations in the highest resolution and accuracy. Secondly, by direct genome-wide analysis of genotypes, epigenetic changes, and gene expression alterations, it is relatively easier to find genetic and epigenetic associations related to a particular phenotype, i.e., for example, antimicrobial resistance. In the isogenic pair of PB367 and PB350, we identified a single and sole mutation, G183D of AmpC. Previously, the G183D substitution in *P. aeruginosa* greatly increased CZA MIC, from 8 to 128 [14] and from 1 to ≥ 8 mg/L [15]; as well as, C/T MIC, from 1 to 32 [13] and from 1/4 to ≥ 16 mg/L [15]. These results were comparable to ours, i.e., PB350 with D183 AmpC was resistant to CZA (MIC = 64 mg/L) and C/T (MIC = 128 mg/L), whereas PB367 with G183 AmpC was susceptible to both CZA and C/T (MICs = 4 mg/L). Therefore, we suggest that G183D mutation is most likely associated with PB350 resistance to CZA and C/T. However, the above-mentioned G183D *P. aeruginosa* isolates demonstrated variable susceptibility to P/T, with either increased MIC (from 4 to 16 mg/L) [15], no change (MIC = 256 mg/L) [14], or decreased MIC, from 128 to 8 [13] and from > 64 to 16 mg/L, in this study (Table 1). We, thus, suspect that there exists an additional, likely epigenetic, factor for *P. aeruginosa* susceptible and resistance to P/T. 

Environmental cues and subinhibitory concentrations of antimicrobial drugs could induce changes in the genomic DNA methylation and modification, followed by alterations in the gene expression pattern of *P. aeruginosa*. In the comparative methylome analysis of PB367 and PB350, we identified two methylation motifs, CT^m6^AC(N)_5_GGG/C^m4^CC(N)_5_GTAG and AAG^m6^AYC, and their putative corresponding MTases belonging to Type I and Type IIG RM systems, respectively. The activity and specificity of these putative MTases require further verification. Notably, the ST235-specific Type I R-M system differs from the one in PAO1 that is responsible for only m6A methylation in G^m6^ATC(N)_6_GTC/G^m6^AC(N)_6_GATC motif [11], indicating a strain-specific immune protection mechanism in *P. aeruginosa*. Nevertheless, the significant epigenetic difference between PB367 and PB350 (Table 2 and Figure 2A) largely relied on m4C methylation and other modifications, and mainly did not come from these two RM systems. This suggests there are other methylation and modification mechanisms differentiated in isolates PB367 and PB350, waiting to be discovered. Regrettably, in the genome-wide gene expression analysis of in vitro cultured PB367 and PB350 isolates we did not unveil any phase-variable MTase being differentially modulated. Future transcriptomic profiling with inclusion of dynamic environmental conditions or stimuli of antimicrobial drugs could facilitate novel findings. 

Despite the vast variable methylation and modification in genomes, we only found 14 genes (13 coding and one tRNA) with significant differential expression between PB367 and PB350. Combinatorial analysis of genomic modification and gene expression revealed only *tox*R (CWI20-24510) had both significantly lowered expression in PB350 and differentially epigenetic modified promoter region. ToxR is a membrane-associated regulatory transcription factor binding to *P. aeruginosa* RNA polymerase and facilitating exotoxin A gene (*tox*A) expression [24]. Although we did not observe significant downregulation of ToxA along with ToxR, the other 12 coding genes with significant differential expression were most likely regulated by ToxR, because we did not observe significant differential methylation or modification in the promoter of these individual genes. Of the 13 methylation-modulated proteins, the outer membrane porin OpdQ, a member of the OprD porin family, became the candidate most responsible for antimicrobial resistance. Previously, OprD porin has been shown to serve as a channel for the β-lactam antibiotic imipenem, whereby mutation or loss of OprD can lead to imipenem resistance [21,22,23]. In addition, OpdQ porin has been shown to be regulated by specific environmental cues associated with cystic fibrosis microenvironment, such as oxygen, nitrate, and NaCl levels [25]. Considering these together, we proposed a model for a methylation-regulated antimicrobial resistance (Figure 3B). Notably, due to the lack of small RNA profiling, as well as small RNA annotation of the genome, differential expression of small RNAs impacted by DNA methylation was not investigated and any small RNA-facilitated gene expression regulation was not taken into account. Therefore, our proposed methylation-induced epigenetic regulatory mechanism is speculative and requires further experimental verifications.

In summary, we explored an isogenic pair of MDR *P. aeruginosa* clinical isolates with integrative genome-wide analyses. We disclosed multiple potential mechanisms of antimicrobial resistance in *P. aeruginosa*, including chromosomal gene mutation (G183D substitution in AmpC), horizontal gene transfer (class 1 integron mobile genetic element likely acquired from plasmids of other species, such as *K. pneumoniae* and *E. coli*), and epigenetic regulation of gene expression (higher OpdQ expression putatively caused by m6A methylation in *tox*R promoter). These genomic and epigenomic changes were more or less associated with bacterial phenotypic alterations in antimicrobial resistance and could have been driven by the use of antimicrobial drugs or other medications, evolutionarily or selectively, which deserves further investigation.

## 4. Materials and Methods

### 4.1. Clinical P. Aeruginosa Isolates and Antimicrobial Susceptibility Tests

Tracheal aspirate and sputum specimens from a 73-year-old female patient were received on or about 3 and 17 April, 2016, respectively, in the Clinical Microbiology Laboratory at the Westchester Medical Center, a tertiary-care hospital in New York. After routine microbiology culture and tests, a pair of *P. aeruginosa* isolates, PB367 and PB350, were identified in the specimens, respectively. Antimicrobial susceptibility tests were performed for a wide array of antimicrobial agents using both the MicroScan Walk-Away 96 system (Beckman Coulter, Brea, CA, USA) and E-test (bioMérieux, Durham, NC, USA). Clinical and Laboratory Standards Institute (CLSI) guidelines (M100-S26) [26] were used to determine antimicrobial susceptibility. Methods carried out were in accordance with the Department of Health and Human Services CFR 45 Part 46 Protection of Human Subjects. The Institutional Review Board of New York Medical College granted a waiver for informed consent from the patient and approved this study.

### 4.2. Whole-Genome Sequencing

Genomic DNA was extracted from each isolate using a QIAamp DNA kit (Qiagen, Frederick, MD, USA) and subjected to both short- and long-read massively parallel sequencing. Short-read sequencing (150 bp × 2) was performed in the NextSeq system using the Nextera XT sample preparation kit (Illumina, San Diego, CA, USA). Genome coverage was reached at ~24× (PB367) and ~20× (PB350) on average. Long-read sequencing was performed using the PacBio RSII SMRT sequencing system. The SMRTbell library was processed using g-TUBE fragmentation (Covaris, Woburn, MA, USA), the BluePippin system for size selection of DNA fragments ranged from 7 to 50 kb (Sage Science, Beverly, MA, USA), and the SMRTbell template preparation kit (PacBio, Menlo Park, CA, USA). Genome coverage was reached at ~110× (PB367) and ~125× (PB350) in average.

### 4.3. De Novo Genome Assembly

PacBio long reads were initially de novo assembled into contigs using the Hierarchical Genome Assembly Process (HGAP) and Quiver tools in SMRT Analysis (v.2.3.0, PacBio). The resulting contigs were trimmed and circularized into a complete chromosome using the Basic Local Alignment Search Tool (BLAST) [27] to identify overlapping sequences. The assembled genome was used as a reference and Illumina short reads were aligned and mapped by both BWA [28] and Bowtie2 [29], followed by sorting and indexing using SAMtools [30]. The alignment and mapping were visualized using Integrative Genomics Viewer (IGV) [31], and the final whole-genome sequences were manually polished and corrected. Both PB367 and PB350 genomes were submitted to the NCBI GenBank (accession numbers: CP025056.3 and CP025055.2) and annotated by both the PATRIC Genome Annotation Service using the RAST tool kit [32,33] and the NCBI Prokaryotic Genome Annotation Pipeline (PGAP). 

### 4.4. Genome Modification Detection and Motif Analysis

The “RS_Modification_and Motif_Analysis.1” protocol in SMRTPortal (version 2.3, PacBio) was used for genome-wide detection of base modifications and identification of specific sequence motifs. PB367 genome was used as a reference, and standard parameters were applied with a minimum modification QV of 30. The REBASE [10] was searched for candidate DNA MTase or RM genes responsible for methylation of a particular sequence motif.

### 4.5. Comparative Genome Analyses

PubMLST (https://pubmlst.org/) was used for multilocus sequence typing (MLST). ResFinder (v2.1) [34] was used for identification of antimicrobial resistance genes. *P. aeruginosa* ST235 complete genomes were screened out and downloaded from the NCBI genome database, with strains PAO1 (NC_002516.2) and AR_0095 (CP027538) selected as controls. ProgressiveMauve multiple genome aligner [35] was used to compare the whole genome sequences. The k-mer weighted inner product, kWIP [36], was used for phylogeny analysis and alignment-free, k-mer-based bacterial relatedness analysis.

### 4.6. Comparative Transcriptomic Analysis Using RNAseq

Total RNA was extracted from bacteria cultured in LB medium at 37 °C after reaching OD_600_ of 0.5. Both isolates, PB367 and PB350, were triplicated. RNA quality was examined by the Bioanalyzer 2100 (Agilent Technologies, Santa Clara, CA, USA) and RNA concentration was determined by Qubit Fluorometric Quantitiation (Thermo-Fisher). After depletion of ribosomal RNA (rRNA) using the Ribo-Zero rRNA Removal Bacteria Kit (Illumia), the RNA was subjected to cDNA sequencing library construction using the TruSeq Stranded mRNA Library Preparation Kit (Illumina) in accordance with the manufacturer’s protocol. Paired-end sequencing (75 bp × 2) was performed in the NextSeq 550 system, reaching more than 17 million reads per sample. EDGE-pro [37] was used to estimate genome-wide gene expression, with the de novo assembled PB367 genome and NCBI PGAP annotation as reference. Differential gene expression was determined using the DESeq2 [38] algorithm, in which a Benjamini–Horchberg adjusted *p*-value less than 0.05 was defined as statistical significance.

## Figures and Tables

**Table 1:** Antibiotic characteristics of two *P. aeruginosa* isogenic isolates from the same patient.

Antibiotic	PB367		PB350	
	MIC (mg/L)	Int	MIC (mg/L)	Int
Amikacin	≤ 16	S	≤ 16	S
Aztreonam	> 16	R	16	I
Ceftazidime	> 16	R	> 16	R
Ceftazidime/avibactam *, #	4	S	64	R
Ceftolozane/tazobactam *	4	S	128	R
Ciprofloxaxin	> 2	R	> 2	R
Cefepime	> 16	R	> 16	R
Gentamicin	> 8	R	> 8	R
Levofloxacin	> 4	R	> 4	R
Meropenem *	> 32	R	16	R
Meropenem/vaborbactam *, #	32	R	16	R
Piperacillin/tazobactam	> 64	R	16	S
Ticarcillin/clavulanate	> 64	R	> 64	R
Tobramycin	> 8	R	> 8	R

MIC, minimum inhibitory concentration (mg/L); Int, interpretation; S, susceptible; I, intermediate; R, resistant; as per CLSI M100-S26 (2016). *, MIC was determined by E-test and #, interpretative criteria were based on FDA-approved drug package insert.

**Table 2:** Genomic modifications and methylation motif sequences in two *P. aeruginosa* isogenic isolates from the same patient.

Modification/Motif	Genome Sites	PB367	PB350	Shared	PB367, not PB350	PB350, not PB367
^m6^A		8850	9032	8659	191 (2.2%)	373 (4.1%)
^m4^C		3467	3810	990	2477 (71.4%)	2820 (74.0%)
other		49097	52337	5204	43894 (89.4%)	47133 (90.1%)
Total		61414	65179	14853	46562 (75.8%)	50326 (77.2%)
AAG^m6^AYC	7918	7913 (99.9%)	7918 (100%)	7913 (99.9%)	0	5
CT^m6^AC(N)_5_GGG	706	705 (99.9%)	706 (100%)	705 (99.9%)	0	1
C^m4^CC(N)_5_GTAG	706	637 (90.2%)	673 (95.3%)	614 (87.0%)	23	59

Note: Y = C + T; N = A + C + G + T.

## Supplementary Materials

Supplementary materials can be found at https://www.mdpi.com/1422-0067/21/3/1026/s1.
